# Long-term evaluation of air sensor technology under ambient conditions in Denver, Colorado

**DOI:** 10.5194/amt-11-4605-2018

**Published:** 2018

**Authors:** Stephen Feinberg, Ron Williams, Gayle S. W. Hagler, Joshua Rickard, Ryan Brown, Daniel Garver, Greg Harshfield, Phillip Stauffer, Erick Mattson, Robert Judge, Sam Garvey

**Affiliations:** 1 Oak Ridge Institute for Science and Education, Oak Ridge, TN 37830, USA; 2 U.S. Environmental Protection Agency (EPA), Office of Research and Development, Research Triangle Park, NC 27711, USA; 3 U.S. EPA Region 8, Denver, CO 80202, USA; 4 U.S. EPA Region 4, Atlanta, GA 30303, USA; 5 State of Colorado Department of Public Health and Environment (CDPHE), Denver, CO, USA; 6 U.S. EPA Region 1, Boston, MA 02109, USA; 7 Jacobs Technology, Inc, Research Triangle Park, NC 27709, USA

## Abstract

Air pollution sensors are quickly proliferating for use in a wide variety of applications, with a low price point that supports use in high-density networks, citizen science, and individual consumer use. This emerging technology motivates the assessment under real-world conditions, including varying pollution levels and environmental conditions. A seven-month, systematic field evaluation of low-cost air pollution sensors was performed in Denver, Colorado, over 2015–2016; the location was chosen to evaluate the sensors in a high-altitude, cool, and dry climate. A suite of particulate matter (PM), ozone (O_3_), and nitrogen dioxide (NO_2_) sensors were deployed in triplicate and were collocated with federal equivalent method (FEM) monitors at an urban regulatory site. Sensors were evaluated for their data completeness, correlation with reference monitors, and ability to reproduce trends in pollution data, such as daily concentration values and wind-direction patterns. Most sensors showed high data completeness when data loggers were functioning properly. The sensors displayed a range of correlations with reference instruments, from poor to very high (e.g., hourly-average PM Pearson correlations with reference measurements varied from 0.01 to 0.86). Some sensors showed a change in response to laboratory audits/testing from before the sampling campaign to afterwards, such as Aeroqual, where the O_3_ response slope changed from about 1.2 to 0.6. Some PM sensors measured wind-direction and time-of-day trends similar to those measured by reference monitors, while others did not. This study showed different results for sensor performance than previous studies performed by the U.S. EPA and others, which could be due to different geographic location, meteorology, and aerosol properties. These results imply that continued field testing is necessary to understand emerging air sensing technology.

## 1 Introduction

Next-generation air monitoring (NGAM) is a quickly evolving and expanding field. Low-cost air pollution sensors have improved the access for both citizens and researchers to obtain pollutant concentration data in more locations. Many new sensors are now sold and marketed to consumers and come with messaging on implications for health. In addition to improving the accessibility of measurement data, air pollution sensors have been used to supplement ambient air monitoring by providing measurements with high spatial density and high time resolution (Mead et al., 2013; Snyder et al., 2013; Kaufman et al., 2017). Low-cost air pollution sensors have the potential to be important enablers of smart cities and the Internet of things (IoT), especially in terms of forecasting and health messaging in megacities with significant variability in microenvironments (Mead et al., 2013; Kumar et al., 2015; Ramaswami et al, 2016). Sensors also enable new techniques for mobile monitoring (McKercher and Vanos, 2018; Woodall et al., 2017). However, without a proper understanding of sensor data quality and calibration, low-cost sensors have the potential to mislead interested community and research groups (Rai et al., 2017). Evaluating how well these sensors perform in both laboratory and field environments is critical for understanding their possible uses in research, citizen science, and consumer use, for individual exposure assessment.

Low-cost air pollution sensors, with purchase prices ranging from the low hundreds to the low thousands of dollars per pollutant, have been developed for both particulate and gas-phase pollutants, including ozone (O_3_) and nitrogen dioxide (NO_2_). Particulate matter (PM) sensors typically measure particle counts using light scattering principles. By using light scattering to measure an ensemble of particles, sensors can be produced that are miniaturized, have a lower cost, and provide real-time data. However, this detection approach can result in bias and inaccuracy from measurement artifacts (Gao et al., 2015; Holstius et al., 2014). Some sensors, such as the OPC-N2 (AlphaSense), measure single particles and allocate them into size bins. This approach is subject to measurement artifacts due to humidity effects and potential particle coincidence, and it assumes particles are spherical and of a homogenous density (Mukherjee et al., 2017). Gas-phase sensors produce a signal through the reaction of the target gases with electrochemical or metal oxide sensors. However, the reactive agents used in these types of sensors may degrade over time, and measurement artifacts may also exist, such as cross-interferences and impacts of temperature (Rai et al., 2017). Therefore, it is necessary to evaluate sensor performance in long-term, real-world study conditions (Lewis and Edwards, 2016; Williams et al., 2014).

The evaluation of low-cost air pollution sensors and their performance is continually evolving (McKercher et al., 2017). Many sensors are evaluated in laboratory settings by exposure to known concentrations of gases and PM, with PM often being evaluated by well-defined aerosols, such as polystyrene latex, in controlled conditions (Wang et al., 2015; Lewis et al., 2016; Manikonda et al., 2016). In outdoor field settings, sensors are often evaluated to determine their performance in comparison with reference methods (Borrego et al., 2016; Jiao et al., 2017; Crilley et al., 2018; Mukherjee et al., 2017; Hagan et al., 2018). Correlations of low-cost sensors have been found to vary from study to study, spanning from negligible to high correlations. Recent studies have shown the correlation between sensors and reference measurements can be improved by the application of correction factors for environmental conditions such as relative humidity (Crilley et al., 2018) or multivariate models and machine learning (Cross et al., 2017; Zimmerman et al, 2018; Hagan et al., 2018).

There are relatively few efforts that exist to systematically examine air pollution sensor technology performance that test a variety of replicate sensor types against reference monitors in a real-world environment. In the United States, the U.S. EPA and the South Coast Air Quality Management District (SCAQMD) have developed field- and laboratory-testing programs for both gas and particulate matter sensors. These efforts represent specific geographic locations and concentration ranges (U.S. EPA, 2017; SCAQMD, 2017). For example, EPA’s Community Air Sensor Network (CAIRSENSE) project tested a variety of gas-phase and particulate-matter sensors in Atlanta, Georgia, under conditions that were high temperature, high humidity, and fairly low ambient concentrations (e.g., hourly PM_2.5_ ranging 0 to 40 μg m^−3^) (Jiao et al., 2016). The SCAQMD AQ-SPEC program similarly conducts field testing of sensor technology in Diamond Bar, California, at a near-road location approximately two months. Evaluation of identical sensors by the EPA and SCAQMD has revealed that the sensor performance may vary by geographical region. For example, Jiao et al. (2016) found AirBeam sensor correlations to be moderate (*r*^2^ ≈ 0.43), while SCAQMD (2017) reported much stronger correlations (*r*^2^ ≈ 0.74). This might be a result of both different concentration ranges as well as the optical properties of the aerosol being measured.

The Community Air Sensor Network (CAIRSENSE) project was a multi-year, multi-location project that focused on evaluating performance characteristics and limitations of low-cost sensors. A prior CAIRSENSE study in Atlanta, Georgia, was conducted in 2014 and early 2015 (Jiao et al., 2016). Atlanta was chosen to test the sensors’ performance in the face of higher temperatures and humidity. For the second part of the CAIRSENSE study, Denver, Colorado, was chosen to test the sensors’ performance under conditions of high altitude, dryness, and lower temperature. Beyond assessing sensor performance through correlation with a reference monitor, this study also investigates the degree to which data from sensors are able to produce similar temporal, wind-direction, and transient-event trends in comparison to high-time-resolution reference monitors.

## 2 Methods

Sensors for this study were selected based on cost, commercial availability, market prevalence, capability, and applicability to EPA research objectives. Table 1 lists the sensors chosen for this study, pollutants measured by each sensor, and the measurement principle used by each sensor. Cost information for these sensors can be found on the EPA’s Air Sensor Toolbox (U.S. EPA, 2017). Two different Dylos units were used for this study. Unit 1 was a Dylos DC1100, while units 2 and 3 were Dylos DC1100 Pro models, where the Pro models are advertised to have increased sensitivity for smaller particles. The Shinyei, Dylos, AirBeam, Aeroqual, and CairClip sensors were used in both the Denver and Atlanta studies (Jiao et al., 2016). Additionally, several of these sensors have been evaluated in laboratory or short-term ambient settings (e.g., Air Sensor Toolbox reference; Sousan et al., 2016; SCAQMD 2017).

Air pollution sensors were acquired and deployed in triplicate. Before deployment, laboratory sensor response audits were performed for all of the available sensors. PM sensors were zero-checked in a clean room environment, all reporting < 2 μg m^−3^ values under those conditions, except for the AirAssure. The software for the AirAssure performs its own zeroing; therefore, they were operated “as is”. A pre-deployment sensor response audit was not performed for the TZOA as it was received shortly before deployment. Sensor output was not adjusted based on the calibration audits in order to reflect their “out of the box” performance. Sensor responses were also audited by either recording their responses to known concentrations (Aeroqual and CairClip sensors) or in a clean air environment (PM sensors) after the end of the measurement period, to evaluate possible sensor drift. Laboratory audit results are presented in the Supplement.

**Figure 1.**
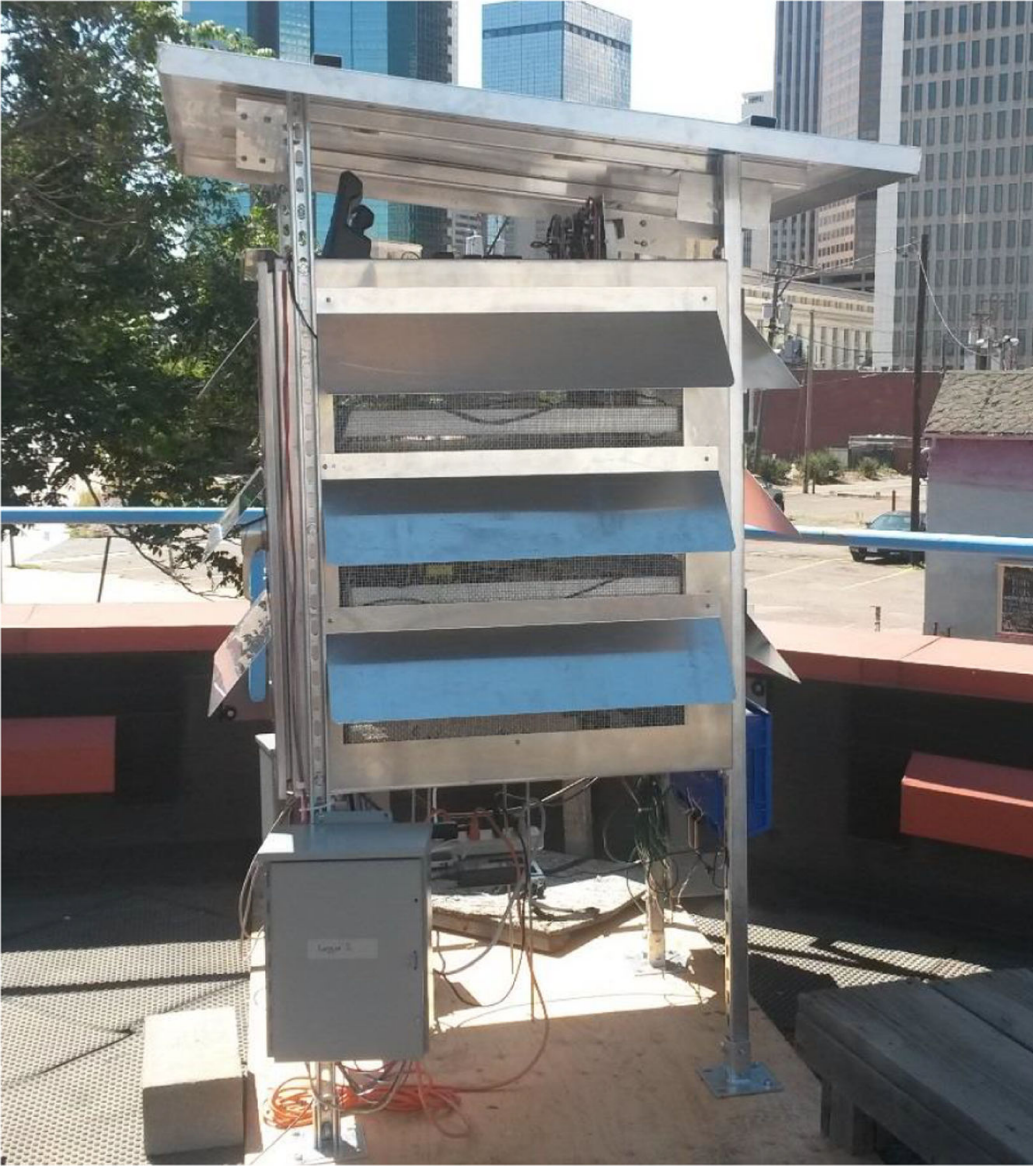
Sensor deployment shelter.

Sensors were deployed at the downtown Denver Continuous Ambient Monitoring Program (CAMP) regulatory monitoring site (latitude: 39.751184; longitude: −104.987625) from September 2015 to March 2016. The CAMP site was operated by the state of Colorado for the duration of the study. Sensors were placed in a ventilated, multi-level shelter designed to allow ambient air circulation and prevent intrusion from precipitation, as shown in Fig. 1. A full description of the shelter has been previously reported (Jiao et al., 2016). The sensors were connected to data loggers stored in weatherproof enclosures attached to the bottom of the shelter. Most of the sensors were connected to Arduino (single-board) microprocessors with either Ethernet (IEEE 802.3 standard) or Recommended Standard 232 (RS-232) serial communication cables. The OPC-N2 and Speck sensor data were logged using laptops, and the TZOA data were stored internally on secure digital (SD) cards. To comply with EPA data security requirements, the cloud based storage capability of the AirAs-sure sensors was disabled, and these units reported data locally via the Arduino microprocessors with onboard memory. The CairClip sensor measures the combined signal from NO_2_ and O_3_. Therefore, both NO_2_ and O_3_ measurements from the CairClip were determined by subtracting the opposite (col-located) reference measurement. The Dylos units also measure multiple particle size fractions. In this study, the “small” particle size fraction, as described by the manufacturer, was used for PM_2.5_ comparisons. TZOA sensors did not have a real-time clock and only measured time as the elapsed number of milliseconds since the device was powered on. Therefore, field operators were required to accurately record start and end times as a means of establishing the sensor response time series.

A total of four Arduino microprocessors and three laptops were used simultaneously for data logging. Between the data loggers, laptops and onboard data storage, there were many different sensor data output formats. Separate data scripts were developed to process each different data format into similarly formatted files for each air pollution sensor type. Once data collections were initiated in September 2015, the sensors were operated with little or no intervention through the entirety of the study. Noted interventions included restarting data systems when they “locked up” or removing snow from the shelves housing the sensors during a major winter snowstorm.

Federal equivalent method (FEM) measurements at the Denver monitoring site were collected using a Teledyne 400E O_3_ monitor, Teledyne 200EU NO_2_ analyzer, and a GRIMM EDM 180 dust monitor, which measured PM_2.5_ and PM_10_ mass at 1 min intervals using optical detection. All sensors and monitors collected pollutant data at 1 min intervals or less. One-minute values were used to generate concentrations at multiple time intervals, with primarily 1 h averages used for data analysis. All averaging and other data processing was performed using the following software: RStudio version 0.98.1103, R version 3.2.2, and the ggplot2, scales, plyr, lattice, corrplot, and “data.table” (extension of “data.frame”) packages.

Sensor data were recovered from the connected laptops and SD cards connected to the data loggers. Most sensors reported data in 1 min intervals. The AlphaSense OPC-N2 units recorded concentrations every 10 s. These measurements were used to calculate 1 min averages. The TZOA sensors reported data based on time elapsed from turning on each unit. The start times for each unit and total elapsed time for each measurement were combined to generate 5 s time stamps for the TZOA measurements. These values were then used to calculate 1 min averages.

In order to best replicate actual use by non-experts and avoid biasing the results towards a positive direction, minimal screening of data was performed. Quality assurance screening consisted primarily of removing data where there was a clear malfunction of the sensor, such as non-numeric data output, or when a sensor (e.g., CairClip unit 1) became “stuck”, reporting a repeated value (value = 255) for long time spans. These types of errors had previously been identified for the output of this sensor type. The Aeroqual units had significant numbers of measurements that, for some reason, were reported as zero. These were possibly due to the inability of the sensor to detect trace concentrations and were therefore not screened out of the data.

Timestamps for all sensors except the TZOA were recorded in Mountain Standard Time. As previously mentioned, TZOA timestamps were generated by combining the initial recording time and the elapsed time reported by the sensors. One-minute measurements and averages were used to calculate 5 min and hourly averages. Hourly averages were further used to calculate 12 h and daily averages. FEM measurements from the state of Colorado instruments were also recorded at 1 min intervals and averaged in the same manner as the sensor data. Data from all sensors and reference instruments were stored in separate data files and combined based on timestamps for analyses using R scripts.

Sensors were also investigated for how well they replicated different trends in the regulatory monitor measurement data. The trends analyzed included average sensor responses based on time of day and wind direction. In order to evaluate these trends, different normalized sensor responses were used. The normalized average sensor response for the diel (daily, 24 h) patterns was calculated as the average concentration for a given hour divided by the average concentration for the hour beginning at 12:00. The normalized average sensor response for wind direction data was defined as the mean concentration for each 10°wind “bin”, divided by the average concentration of the 170 to 180°bin. The sensor response times were also analyzed by calculating the average 1 min relative sensor response, as defined by the distribution of the 1 min concentration differences divided by the average sensor response.

## 3 Results and discussion

Table 2 shows a summary of data completeness from the air pollution sensors, including the total percentage of minutes measured, percentage of measurements missed by not logging data, and the percentage of completely missing data. The majority of missing data was due to events where the sensor and data loggers were inoperative. The most significant of these events was due to snow intrusion into the monitoring platform in December 2015, which caused units to shut down. Most sensors had a very high data capture rate throughout the study when the units were on (and operational). The CairClip units had significant amounts of missing data, likely due to data transmission errors from the universal asynchronous receiver-transmitter (UART) serial communication system. In the previous Atlanta study as well as in a Newark-based citizen science study (Kaufman et al., 2017), CairClip units with identical sensors but different universal serial bus (USB) data connections were used and did not have significant amounts of missing data.

**Figure 2.**
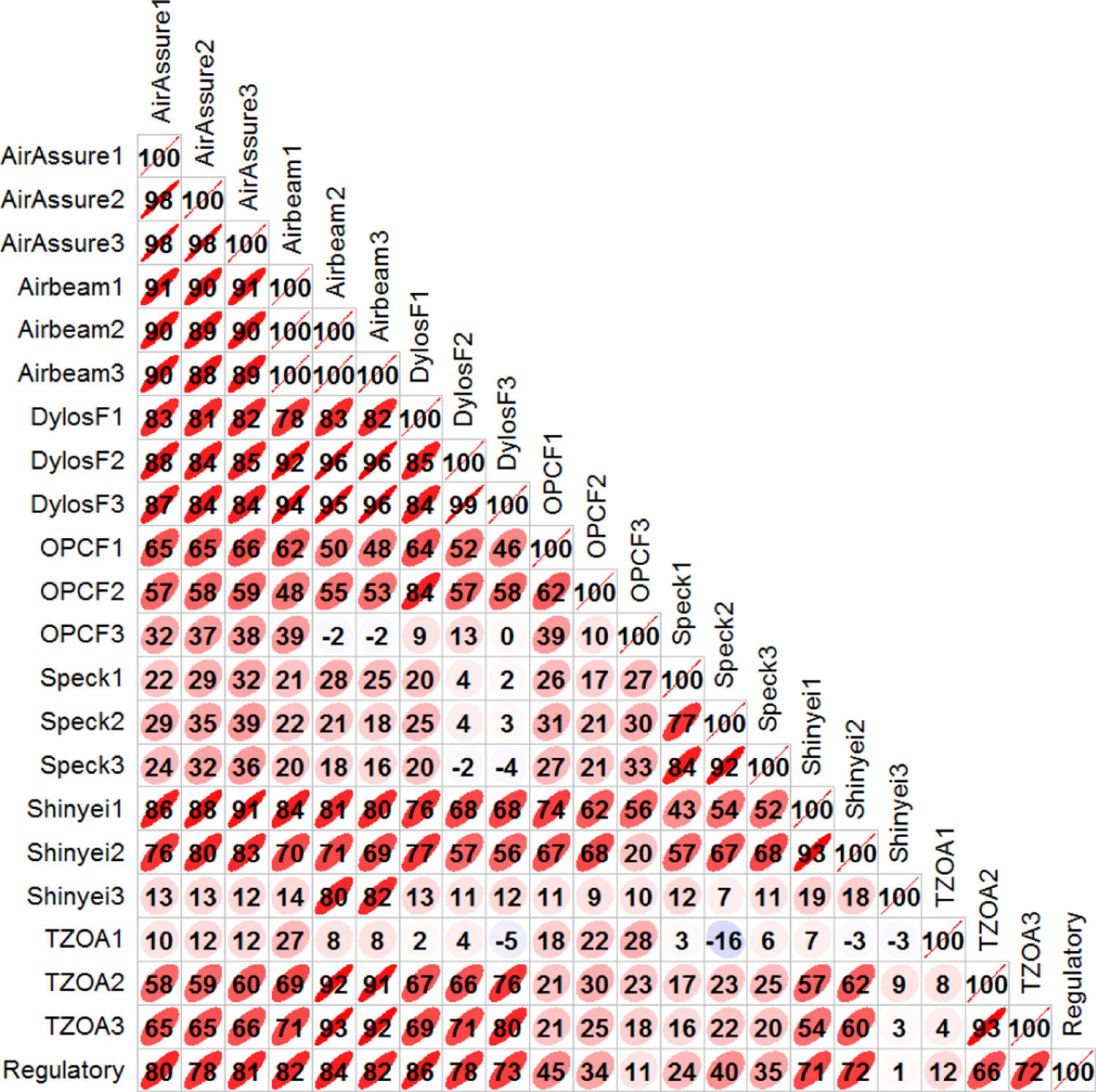
Correlation (*r* × 100) plot for sensors measuring fine PM. Ellipses represent the overall scatter of the data (1 h averaged measurements).

Measurements from air pollution sensors and regulatory monitors were time-averaged at multiple intervals for comparison. The time intervals included 5 min, hourly, 12 h, and daily averages. For each set of time averaging, regressions were calculated to evaluate sensor correlation and bias when compared to regulatory measurements. Additionally, inter-comparisons were made between sensors of the same pollutant type (e.g., correlations between PM sensors). Table 3 displays a summary of regression statistics for sensors when compared to regulatory measurements as well as precision calculations for 1 h time averages. The precision was calculated as the root mean square (rms) of the hourly coefficients of variation. In general, correlations were greatest at the 1 h time average. Correlations in general improved slightly with increasing length of the averaging period up to hourly averages. Reduced correlations for most sensors at the 12 h and daily averages may be a result of a lower number of data points. In contrast to most other measurements, sensors that reported data for coarse PM (Dylos) or PM_10_ (OPC-N2) showed improved correlations with increasing averaging time for those measurements. The correlations for all the time averaging periods can be found in the Supplement. Sensors that measured particle count had better precision than those measuring particle mass concentrations. Figure 2 shows a Pearson correlation (*R*) plot for 1 h average reference (SoC) and PM sensor measurements. The PM units show high correlation among sensors of the same model, except for when one sensor in a group had significant issues. Of the PM_2.5_ sensors, the AirAssure, AirBeam, and Dylos (*R* = 0.73 to 0.86) units exhibited the highest correlation with reference measurements. Dylos unit 1 had the highest linearity; however, it had the lowest particle count response, both of which are likely explained by not detecting the smallest particles as effectively as other units. CairClip unit 1 rarely properly transmitted data throughout the study, leading to its low correlations. CairClip units 2 and 3 had more sporadic data transmission issues. All CairClip units recovered data properly once returned to the lab after the field campaign where their internal data storage was used. The response from Shinyei unit 3 changed in mid-October. The correlation between the unit and the reference monitor was initially 0.01, then increased to 0.84 when comparing only the data starting October 16 and later.

Several sensor models were used in both the Atlanta and Denver CAIRSENSE evaluation campaigns. Both studies deployed the AirBeam, Dylos, and Shinyei PM sensors. In all cases except for Shinyei unit 3, these sensors showed greater linearity in Denver than in Atlanta, when comparing 12 h averages. When only considering data after October 16, Shinyei unit 3 also had higher correlation in Denver than in Atlanta. This may be due to less noise caused by lower humidity in Denver than in Atlanta. Aeroqual and CairClip air pollution sensors were also deployed in both Atlanta and Denver. O_3_ measured by the Aeroqual units showed similar correlations in both locations (*R*^2^ = 0.82 to 0.94 in Atlanta, *R*^2^ = 0.85 to 0.92 in Denver). O_3_ measured by CairClip units 2 and 3 in Denver showed poorer correlations than the CairClip units used in Atlanta (*R*^2^ = 0.00 to 0.21 in Denver versus *R*^2^ = 0.68 to 0.88 in Atlanta). However, NO_2_ measured by CairClip units 2 and 3 in Denver was more highly correlated than in Atlanta (*R*^2^ = 0.71 to 0.76 in Denver versus 0.57 in Atlanta).

**Figure 3.**
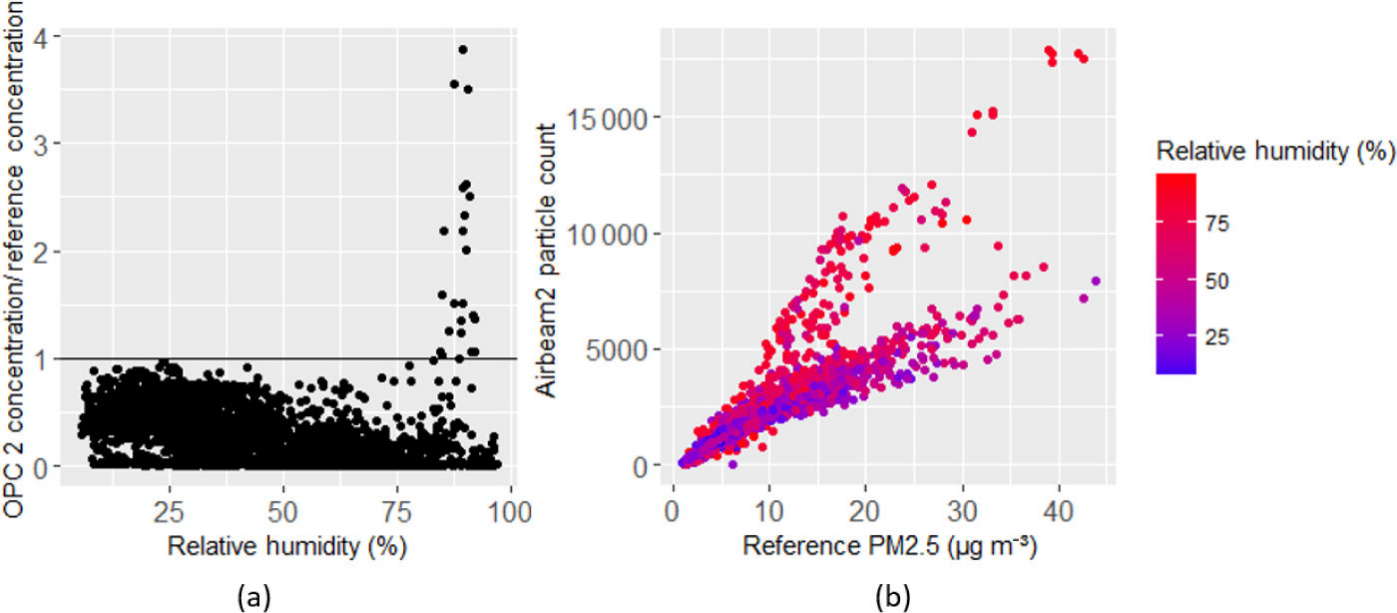
OPC-N2 PM_2.5_ and relative humidity (**a**) and hourly average FEM PM_2.5_ concentration and AirBeam particle count stratified by relative humidity (**b**).

While Denver is not necessarily known for high humidity, humidity artifacts were observed in some sensors. Figure 3a shows the PM_2.5_ concentrations measured by one of the OPC-N2 against relative humidity. At relative humidity around 90 %, the PM concentration spikes significantly, suggesting that humidity is interfering with the sensor response measurement. This behavior is similar to that observed by Sousan et al. (2016). Some other instruments also had different responses based on humidity. Figure 3b shows hourly particle counts measured by an AirBeam sensor against PM_2.5_ concentration measured by the reference instrument, stratified by relative humidity. There appear to be two separate relations between reference measured concentrations and sensor measured particle counts, with a greater particle count response occurring more at higher humidity. This relationship was observed in each of the AirBeam sensors. An example of humidity relationships from each sensor type can be found in the Supplement.

**Figure 4.**
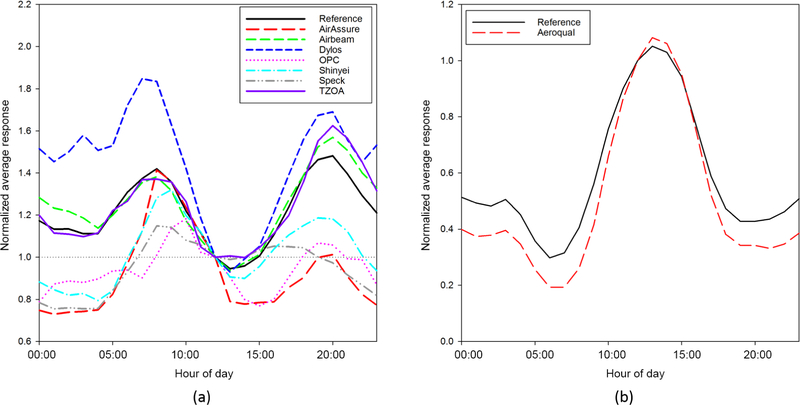
Diel patterns for (**a**) PM_2.5_ and (**b**) O_3_ sensor and reference measurements.

In addition to understanding the precision of air pollution sensors and how well they correlate with reference measurements, it is also important to understand how well a sensor can capture trends and distributions of pollutant concentrations. There are many ways to examine these trends and distributions. Figure 4 shows the diel patterns of PM_2.5_ (a) and O_3_ (b) reference and sensor measurements respectively. The results, for each sensor, represent the measurements of the best performing unit for each sensor type/model, as determined by *R*^2^ values. The various PM air pollution sensors have a wide range of comparisons to the reference monitor. Two sensors (TZOA and AirBeam) show similar patterns throughout the day, while some other sensors do not reflect the reference diel pattern at all (e.g., OPC, Speck). It is interesting to note that both the TZOA and AirBeam measure particle count; however, there is no basis to say why these sensors performed better than those measuring mass concentrations. The Aeroqual sensor diel pattern was similar to that of the reference O_3_ monitor. The nature of the calculation of O_3_ and NO_2_ by subtraction, and missing data from the CairClip sensors, prevented this analysis from providing meaningful results.

**Figure 5.**
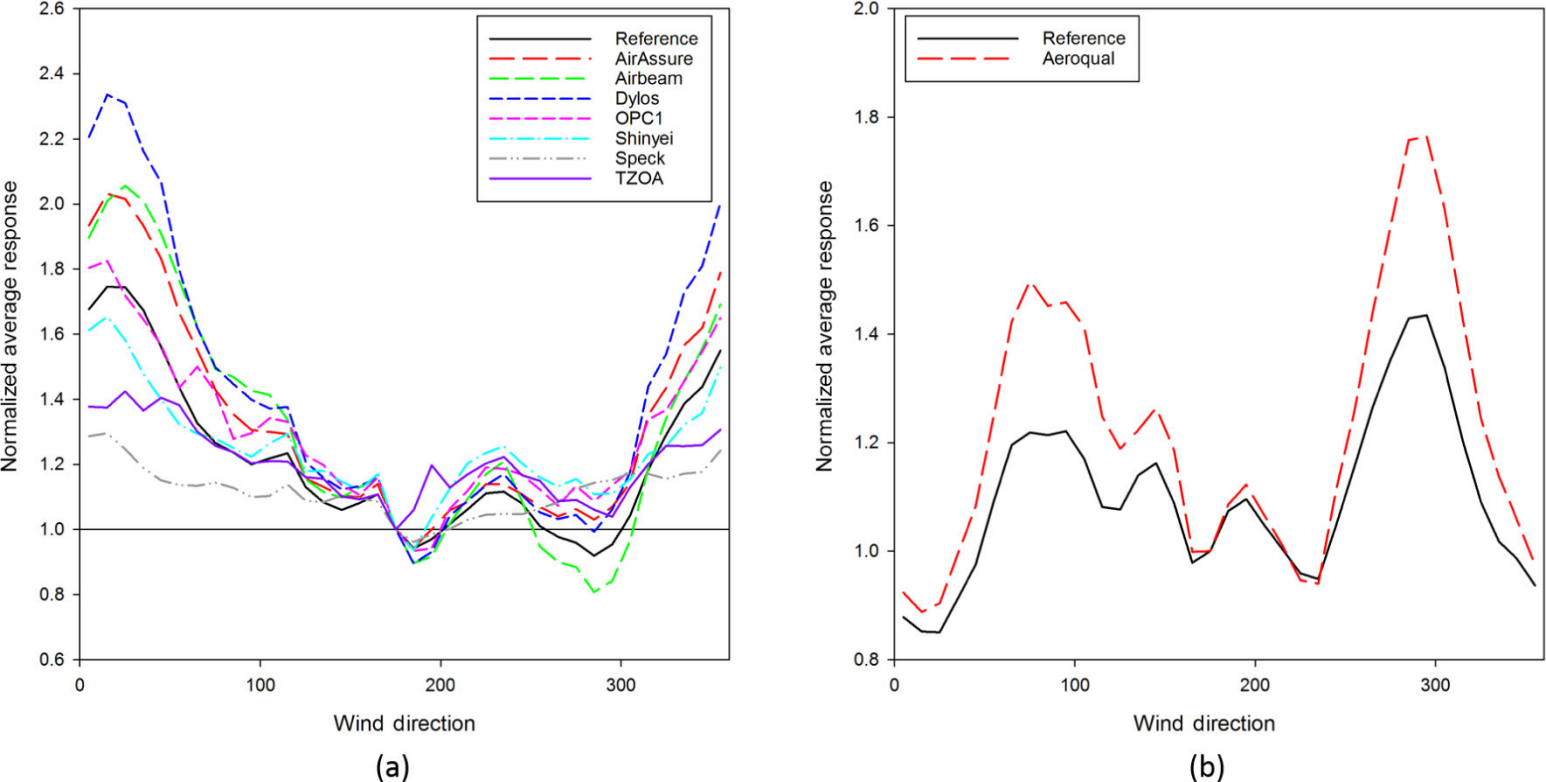
Wind direction patterns for (**a**) PM_2.5_ and (**b**) O_3_ sensor and reference measurements.

Air quality measurements are also known to be dependent on wind direction, and it is important to know whether these differences were reflected in the sensor measurements. Figure 5 shows the normalized average sensor response PM_2.5_(a) and O_3_ (b) response of the sensors and the reference monitors respectively. The reference monitor response is represented by the black line. Both the highest concentrations and greatest variation from the reference monitor concentrations occurred when winds were from the north, where there are multiple large roadways and a railyard. However, there was no other evidence to suggest that these sources contributed to differences in the measurement trends. The sensors generally compared more favorably with the reference monitors when examining the wind direction dependence of concentration. This is most apparent in the OPC-N2 sensor, where the sensor trends track the trends measured by the reference monitor. This increases the confidence that sensors may be useful in studies that pair wind direction with concentration to determine potential bearings or locations of pollution sources to supplement source apportionment and receptor modeling. It also raises questions as to why an air pollution sensor would be able to reproduce wind direction trends but not necessarily reproduce daily concentration measurement patterns. We undertook exploration of this perplexing result, but we were not able to determine a clearly identifiable cause. While relative humidity and temperature do have time-of-day variation that is not reflected in wind direction, we were unable to use these parameters to explain the differences between time-of-day and wind-direction trends.

**Figure 6.**
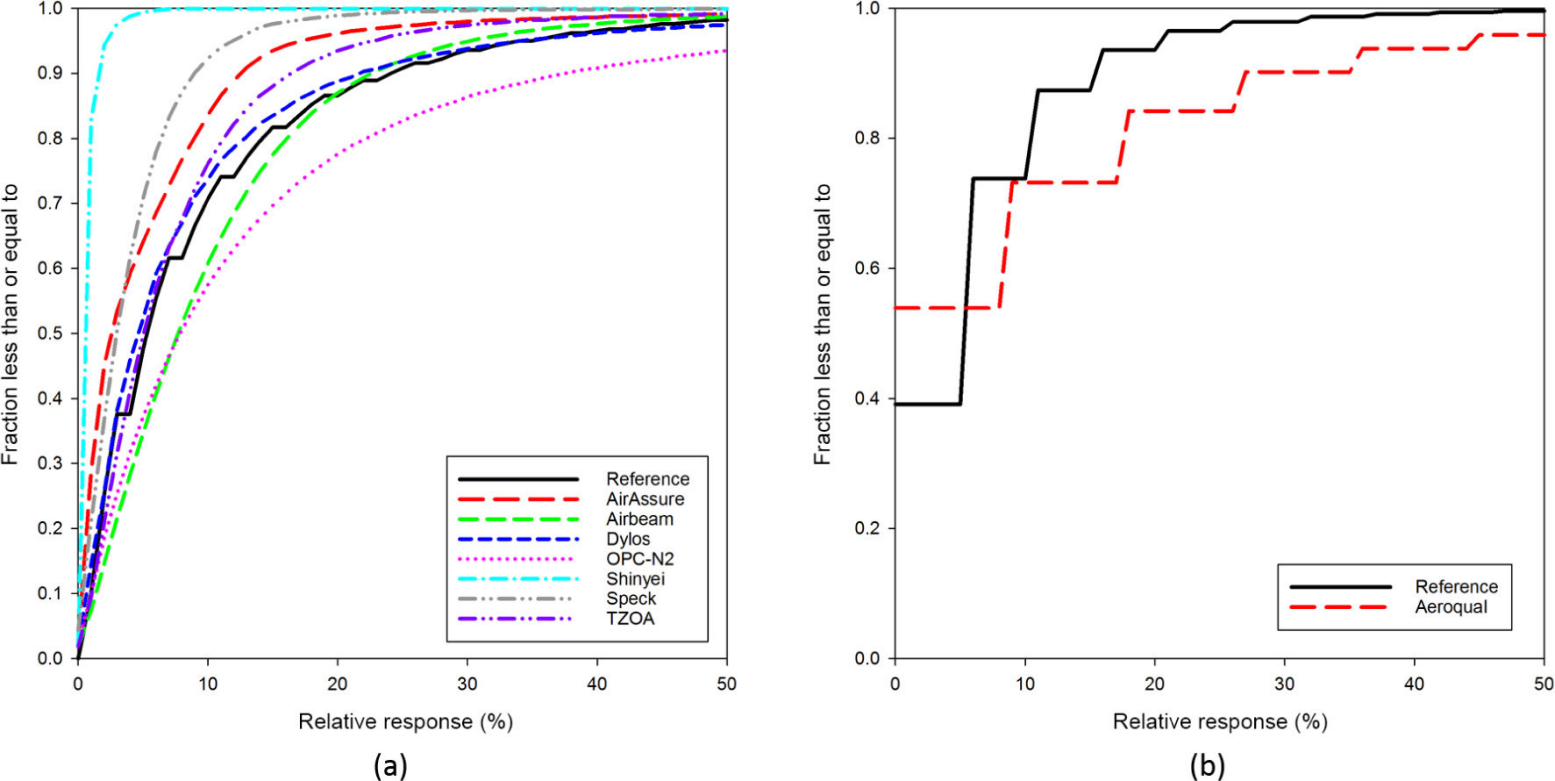
Cumulative distribution functions for 1 min response differences for (**a**) PM_2.5_ and (**b**) O_3_ sensor and reference measurements.

The high-time-resolution data collected for this study allowed for the examination of air pollution sensor response trends compared to that of regulatory air pollution monitors. Figure 6 shows a cumulative distribution function (CDF) for the relative change in sensor and regulatory monitor response between 1 min measurements for PM_2.5_ (a) and O_3_ (b) sensor and reference measurements respectively. The relative response was calculated as the absolute value of the difference between consecutive 1 min measurements divided by the mean measurement over the entire study period for each sensor/monitor. If the reference monitor were considered a perfect measurement, sensor curves to the left and above the reference monitor line would have smaller relative changes than the reference monitor, indicating a slower response to changes in concentration, while curves below and to the right of the monitor line would signify larger measurement-to-measurement changes than the reference monitor, indicating potential high levels of measurement noise. Most PM monitors exhibited a slower response to changes in concentration than the reference monitor. The OPC-N2 and AirBeam sensors were the only ones with curves to the right of the reference monitor, suggesting that they may have more noise in their measurements. The Aeroqual sensor showed more O_3_ measurement noise when compared to the reference measurement.

## 4 Conclusions

Nine different air pollution sensor devices were deployed in triplicate with collocated air pollution reference monitors in Denver, Colorado, over an extended operational timeline of longer than six months. The sensors showed a wide range of correlations with reference measurements, but they tended to have high correlation with sensors of the same model. PM sensors deployed in both Denver and Atlanta had higher correlations with reference monitors in Denver than in Atlanta. This is likely due to less humidity-related response in Denver. Aeroqual O_3_ measurements in Denver showed similar linearity to those measured in Atlanta. CairClip O_3_ correlations were lower in Denver than in Atlanta, but NO_2_ correlations were higher. Sensors that have also been evaluated by the South Coast Air Quality Management District (SCAQMD) tended to show similar results in terms of correlation (SCAQMD, 2017). However, in all cases, sensors’ performance in this long-term field deployment was lower than that of laboratory-based comparisons performed in this study and others (U.S. EPA, 2017). It is not surprising that the results of this study for PM sensors varied from other studies, as the responses to optical measurement techniques used by these sensors are likely influenced by aerosol composition. This study demonstrates the need for long-term, real-world evaluation studies for current and future air pollution sensors, which should be performed in locations with different air pollutant concentration ranges and aerosol characteristics.

Several air pollution sensors were able to capture variations in important trends, such as diel patterns and wind direction dependence on concentration. However, the OPC-N2 units showed similar results to reference monitor measurement data when analyzing the wind direction trends but not when analyzing “time-of-day” trends. These promising results show that sensors have the possibility for supplementing measurement research capabilities when interested in air pollution trends such as those dependent on wind direction. Analyses of wind-direction-based air pollutant trends could be useful for possible identification of source locations or regions, especially with the use of a sensor-based network.

## Supplementary Material

SI

## Figures and Tables

**Table 1.** Sensors used during the CAIRSENSE – Denver study.

Sensor	Pollutant(s) measured	Principle of operation
Aeroqual SM-50	O_3_	Electrochemical sensor
TSI AirAssure	PM	Light scattering
AirCasting AirBeam	PM	Light scattering
Cairpol CairClip	NO_2_ + O_3_	Electrochemical sensor
Dylos DC1100/DC1100 Pro	PM	Laser particle counter
AlphaSense OPC-N2	PM	Laser particle counter
Shinyei PMS-SYS-1	PM	Light scattering
AirViz Speck	PM	Light scattering
TZOA PM Research Sensor	PM	Laser particle counter

**Table 2.** Sensor data completeness.

Sensor	Measurement %	Sensor on and not logging %	Completely missing %	Comments
Aeroqual	82%	0%	18%	45 % of logged values were 0
	73%	0%	27%	42 % of logged values were 0
	81 %	5%	13%	32 % of logged values were 0
	87%	0%	13%	
AirAssure	87%	0%	13%	
	87%	0%	13%	
	74%	0%	25 %	
AirBeam	62%	6%	32%	
	62%	6%	32%	
	29%	53 %	18%	56 % of logged values were 255*
CairClip	63%	13%	24%	No data before 10 Aug 2015
	63 %	23 %	13%	
	82%	0%	18%	
Dylos	82%	0%	18%	
	72 %	1%	27%	
	77 %	0%	23 %	
OPC-N2	76 %	0%	24%	
	71 %	0%	29%	59 % of logged values were 0
	82%	0%	18%	
Shinyei	73 %	0%	27%	
	87%	0%	13%	
	92 %	0%	8%	
Speck	93 %	0%	7%	
	96 %	0%	4%	
	61 %	0%	39%	
TZOA	47 %	0%	53%	
	47 %	0%	53%	

* 255 represented a communication or other unknown sensor failure.

**Table 3.** Regression and precision results for CAIRSENSE sensors (1 h time averaged).

Sensor	Pollutant	Reference average concentration^1^	Slope	Intercept	Pearson correlation, r	rms precision (%)	zumber of hourly measurements
Aeroqual SM-50	O_3_, ppb	18.8 ppb	0.56	−0.004	0.93	73	3325
			0.58	−0.004	0.92		2963
			0.77	−0.004	0.96		3279
TSI AirAssure	PM, μg m^−3^	7.8 μgm^−3^	1.14	2.64	0.8	41	3486
			1.13	−0.04	0.78		3486
			1.19	−1.38	0.81		3486
AirCasting AirBeam	Particle count, hundreds of particles per cubic foot (hppcf)	7.8 μgm^−3^	273	−323	0.82	6	3028
			278	−124	0.84		2539
			322	−352	0.82		2532
Cairpol CairClip	O_3_, ppb	18.8 ppb	NA^2^	NA^2^	NA^2^	NA^2^	738
			−0.04	−23.6	−0.06		2831
			1.03	−39.0	0.46		2852
Cairpol CairClip	NO_2_, ppb	26.8 ppb	NA^2^	NA^2^	NA^2^	NA^2^	738
			0.65	−10	0.87		2831
			0.67	−15	0.84		2852
Dylos DC1100/DC1100 Pro	“Small” particle count, hppcf	7.8 μgm^−3^	64	−152	0.86	15	3324
			428	−1182	0.78		3324
			431	−941	0.73		2937
Dylos DC1100/DC1100 Pro	“Large” particle count, hppcf	12.0 μgm^−3^	1.3	5.5	0.40	10	3324
			5.7	73	0.33		3324
			4.9	84	0.27		2937
AlphaSense OPC-z2	PM_2.5_,μgm^−3^	7.8 μgm^−3^	0.4	−0.30	0.45	108	2969
			0.49	−1.66	0.34		2939
			0.07	0.60	0.11		2735
AlphaSense OPC-z2	PM_10_, μgm^−3^	19.6 μgm^−3^	0.45	2.98	0.47	101	2969
			0.54	−1.06	0.68		2939
			0.12	2.86	0.20		2735
Shinyei PMS-SYS-1	PM_2.5_, μg m^−3^	7.8 μgm^−3^	0.58	0.24	0.71	20	3325
			0.54	0.8	0.72		2963
			0.42	4.35	0.01^3^		3486
AirViz Speck	PM_2.5_, μg m^−3^	7.8 μgm^−3^	0.76	13	0.24	37	3557
			0.74	15	0.40		3584
			0.62	10	0.35		3971
TZOA PM Research Sensor	Particle count, hppcf	7.8 μgm^−3^	NA^2^	NA^2^	NA^2^	17^4^	2341
			6.68	1.37	0.66		1838
			6.75	2.16	0.72		1836

1 Average concentration calculated for hours with valid sampling data.

2 Correlation results not shown due to large amount of missing or invalid data.

3 Shinyei unit 3’s correlation improved to 0.84 when only considering data from October 16 and later.

4 TZOA unit 1 was excluded from rms precision calculations.
