# Supplementary material for: Long-term evaluation of air sensor technology under ambient conditions in Denver, Colorado
Source: Atmos Meas Tech. Author manuscript; Available in PMC 2019 Oct 8. (PMC6781239; doi:10.5194/amt-11-4605-2018)
Supplement: SI [file NIHMS1041048-supplement-SI.pdf]

Supplement of Atmos. Meas. Tech., 11, 4605–4615, 2018
https://doi.org/10.5194/amt-11-4605-2018-supplement
© Author(s) 2018. This work is distributed under
the Creative Commons Attribution 4.0 License.

Atmospheric Measurement Techniques
Open Access

Supplement of

Long-term evaluation of air sensor technology under ambient conditions in Denver, Colorado

Stephen Feinberg et al.

Correspondence to: Stephen Feinberg (feinberg.stephen@epa.gov)

The copyright of individual parts of the supplement might differ from the CC BY 4.0 License.

SUPPLEMENTAL MATERIAL

Table S1 Aeroqual SM-50 Calibration Audit Results

Unit	Pre- Denver Slope	Pre- Denver Intercept	Pre-Denver r^2	Post-Denver Slope	Post-Denver Intercept, ppb	Post-Denver r^2
Aeroqual 1	1.2	1.1	0.9996	0.53	0.25	0.9913
Aeroqual 2	1.2	1.3	0.9998	0.63	-1.00	0.9987
Aeroqual 3	1.2	3.8	0.9997	0.57	2.78	0.993

Table S2 TSI Air Assure Calibration Audit Results

Unit	Pre-Denver Zero (µg/m³)	Post-Denver Zero (µg/m³)
Air Assure 1	5	4.46
Air Assure 2	3	2.54
Air Assure 3	1	0.64

Table S3 AirCasting Airbeam Calibration Audit Results

Unit	Pre-Denver Zero[1]	Post-Denver Zero (hppcf)
Airbeam 1	NA	0
Airbeam 2	NA	0
Airbeam 3	NA	0

[1]Pre-Denver Audits were not performed for Airbeam units

Table S4 Cairpol CairClip Calibration O_3 Audit Results

Unit	Pre- Denver Slope[1]	Pre- Denver Intercept[1]	Pre-Denver r^2	Post- Denver Slope	Post- Denver Intercept, ppb	Post-Denver r^2
CairClip 1	NA	NA	NA	1.22	-0.47	0.9984
CairClip 2	NA	NA	NA	1.03	5.59	0.9994
CairClip 3	NA	NA	NA	1.29	-20.8	0.9991

[1]Pre-Denver Audits were not performed for CairClip units

Table S5 Cairpol CairClip Calibration NO_2 Audit Results

Unit	Pre- Denver Slope[1]	Pre- Denver Intercept[1]	Pre-Denver r^2	Post- Denver Slope	Post- Denver Intercept, ppb	Post-Denver r^2
CairClip 1	NA	NA	NA	0.93	0	0.9993
CairClip 2	NA	NA	NA	0.88	0	0.9941
CairClip 3	NA	NA	NA	1.01	0	0.9988

[1]Pre-Denver Audits were not performed for CairClip units

Table S6 Dylos DC-1100/DC-1100 Pro Calibration Audit Results

Unit	Pre-Denver Zero (particle count)	Post-Denver Zero (particle count)
Dylos 1	0	0
Dylos 2	0	0
Dylos 3	0	0

Table S7 Alphasense OPC-N2 Calibration Audit Results

Unit	***Pre-Denver Zero (µg/m^3)***	***Post-Denver Zero (µg/m^3)***
OPC 1	0	0
OPC 2	0	0
OPC 3	0	0

Table S8 Shinyei PMS-SYS-1 Calibration Audit Results

Unit	***Pre-Denver Zero (µg/m^3)***	***Post-Denver Zero (µg/m^3)***
Shinyei 1	0	0
Shinyei 2	0	0
Shinyei 3	0	0

Table S9 AirViz Speck Calibration Audit Results

Unit	***Pre-Denver Zero (µg/m^3)***	***Post-Denver Zero (µg/m^3)***
Speck 1	0	0
Speck 2	0	10
Speck 3	0	4

Table S10 Correlation Results for CAIRSENSE sensors at different time intervals

Sensor	Pollutant	5-minute	1-hour	12-hour	24-hour
Aeroqual SM-50	O_3, ppb	0.93<br>0.92<br>0.96	0.93<br>0.92<br>0.96	0.91<br>0.98<br>0.97	0.86<br>0.83<br>0.95
TSI Air Assure	PM, μg/m^3	0.78<br>0.77<br>0.80	0.8<br>0.78<br>0.81	0.55<br>0.52<br>0.54	0.39<br>0.37<br>.39
AirCasting AirBeam	Particle Count, hundreds of particles per cubic foot (hppcf)	0.81<br>0.83<br>0.81	0.82<br>0.84<br>0.82	0.82<br>0.83<br>0.80	0.79<br>0.82<br>0.79
Cairpol Cairclip	O_3, ppb	NA[1]<br>-0.42<br>0.40	NA[1]<br>-0.06<br>0.46	NA[1]<br>-0.12<br>0.47	NA[1]<br>0.65<br>-0.42
Cairpol Cairclip	NO_2, ppb	NA[1]<br>0.82<br>0.83	NA[1]<br>0.873<br>0.84	NA[1]<br>0.61<br>0.79	NA[1]<br>0.50<br>0.77
Dylos DC1100/DC1100 Pro	"Small" Particle Count, hppcf	0.85<br>0.77<br>0.72	0.86<br>0.78<br>0.73	0.86<br>0.77<br>0.72	0.86<br>0.75<br>0.70
Dylos DC1100/DC1100 Pro	"Large" Particle Count, hppcf	0.38<br>0.34<br>0.28	0.40<br>0.33<br>0.27	0.57<br>0.34<br>0.27	0.69<br>0.40<br>0.32
Alphasense OPC-N2	$PM_{2.5}$, μg/m^3	0.41<br>0.32<br>0.11	0.45<br>0.34<br>0.11	0.51<br>0.34<br>0.07	0.51<br>0.36<br>0.00
Alphasense OPC-N2	PM_{10}, μg/m^3	0.40<br>0.63<br>0.20	0.47<br>0.68<br>0.20	0.69<br>0.70<br>0.15	0.74<br>0.70<br>0.16
Shinyei PMS-SYS-1	$PM_{2.5}$, μg/m^3	0.71<br>0.70<br>0.01	0.71<br>0.72<br>0.01*	0.69<br>0.71<br>0.05	0.64<br>0.70<br>0.10
AirViz Speck	$PM_{2.5}$, μg/m^3	0.24<br>0.40<br>0.36	0.24<br>0.40<br>0.35	0.21<br>0.38<br>0.31	0.26<br>0.40<br>0.31
TZOA PM Research Sensor	Particle Count, hppcf	NA[1]<br>0.50<br>0.68	NA[1]<br>0.66<br>0.72	NA[1]<br>0.64<br>0.71	NA[1]<br>0.57<br>0.67

[1]Correlations were not performed

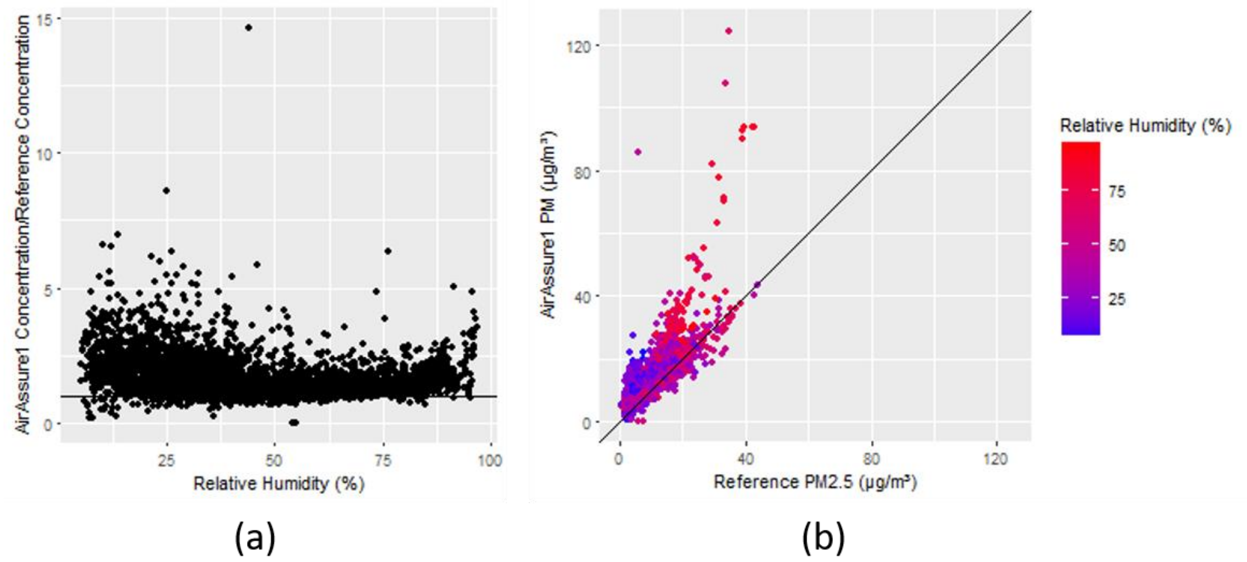

Figure S1 AirAssure1 $PM_{2.5}$ to reference concentration ratio and Relative Humidity (a) and Hourly Average FRM $PM_{2.5}$ concentration and AirAssure1 PM concentration stratified by Relative Humidity (b)

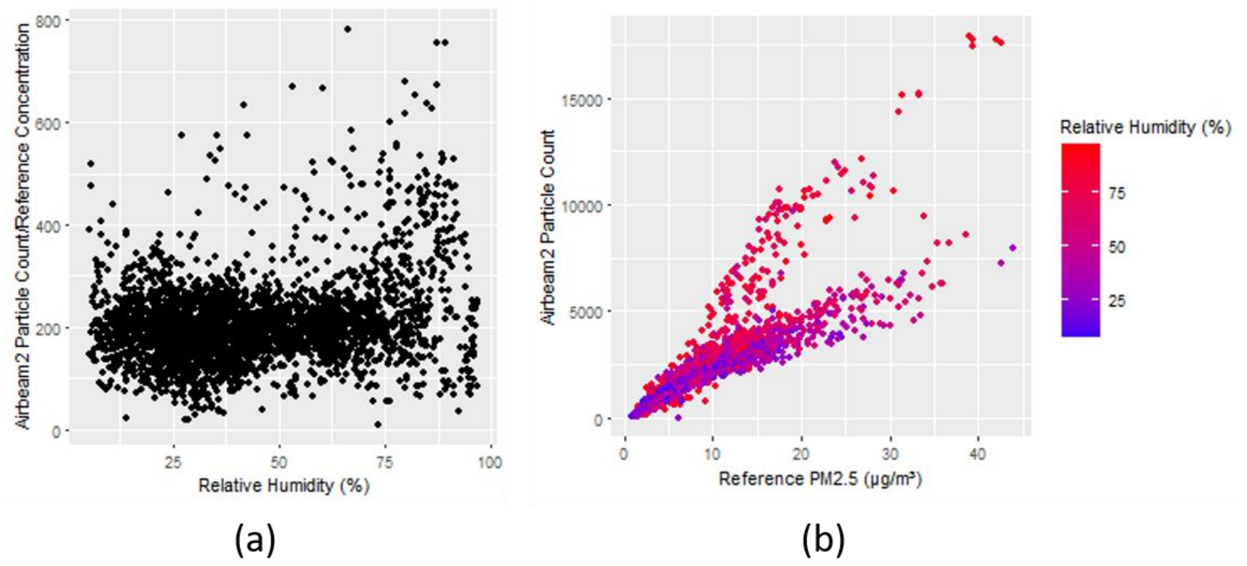

Figure S2 Airbeam2 Particle Count to reference concentration ratio and Relative Humidity (a) and Hourly Average FRM $PM_{2.5}$ concentration and Airbeam2 Particle Count stratified by Relative Humidity (b)

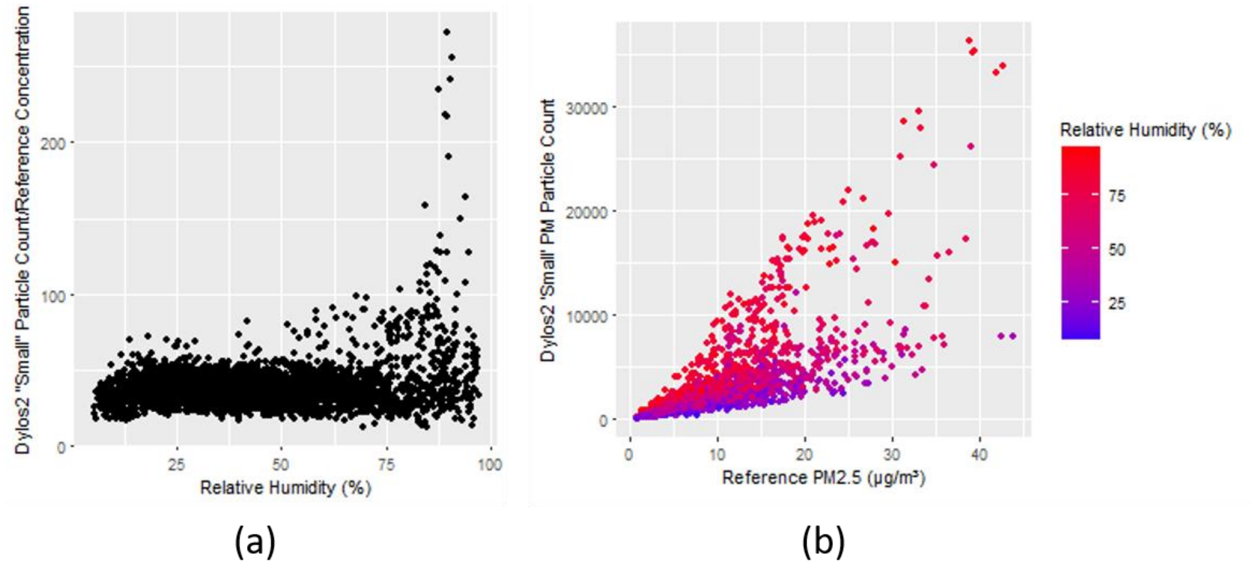

Figure S3 Dylos2 “Small” Particle Count to reference concentration ratio and Relative Humidity (a) and Hourly Average FRM $PM_{2.5}$ concentration and Dylos2 “Small” Particle Count stratified by Relative Humidity (b)

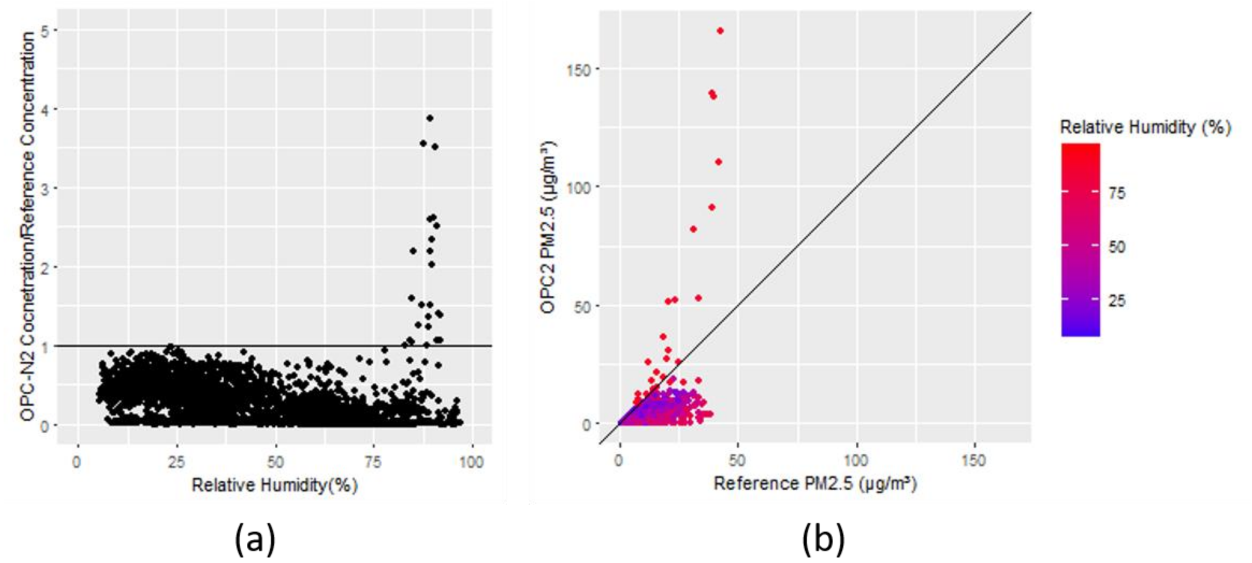

Figure S4 OPC2 $PM_{2.5}$ to reference concentration ratio and Relative Humidity (a) and Hourly Average FRM $PM_{2.5}$ concentration and OPC2 PM concentration stratified by Relative Humidity (b)

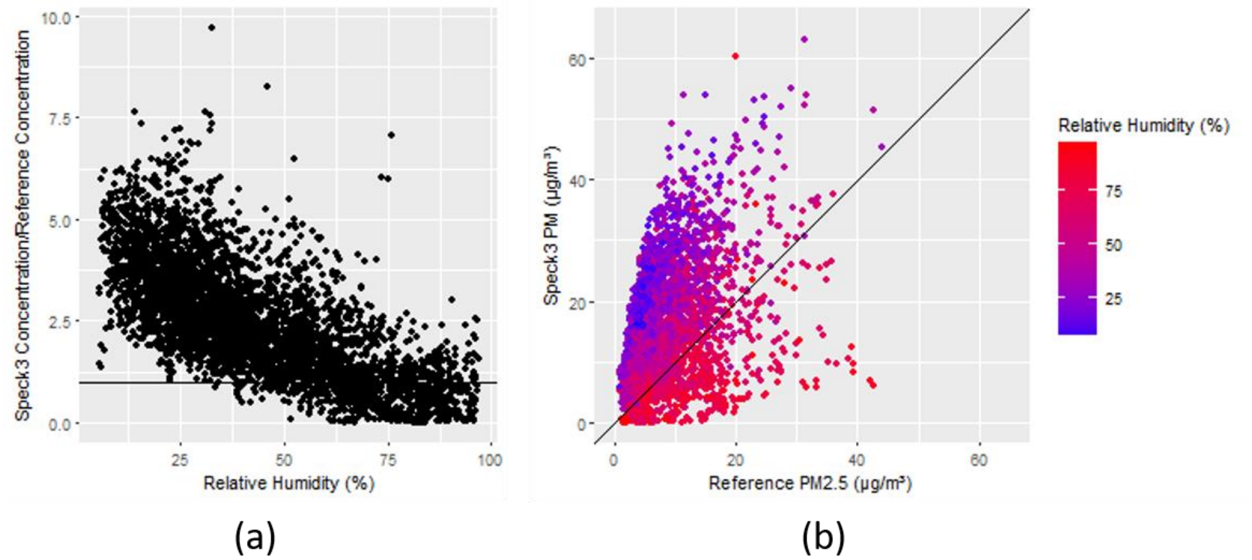

Figure S5 Speck3 $PM_{2.5}$ to reference concentration ratio and Relative Humidity (a) and Hourly Average FRM $PM_{2.5}$ concentration and Speck3 PM concentration stratified by Relative Humidity (b)

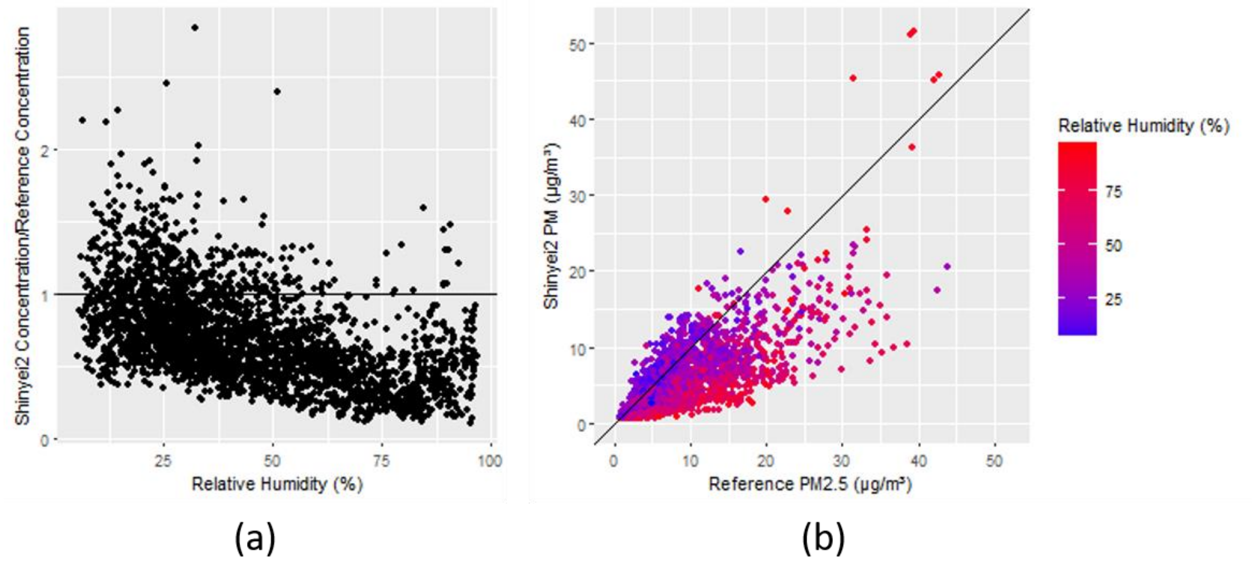

Figure S6 Shinyei2 $PM_{2.5}$ to reference concentration ratio and Relative Humidity (a) and Hourly Average FRM $PM_{2.5}$ concentration and Shinyei2 PM concentration stratified by Relative Humidity (b)

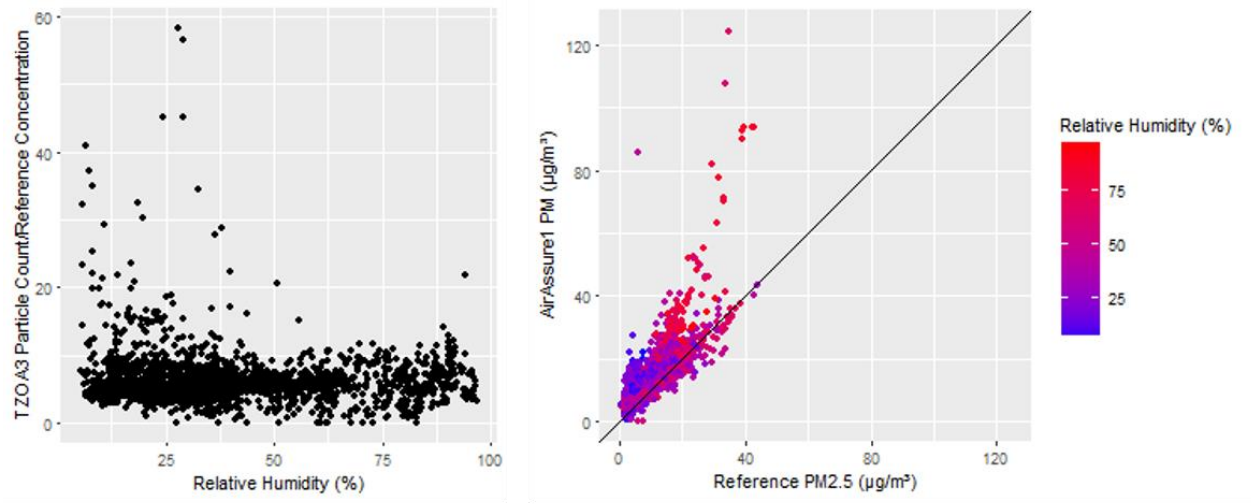

(a) (b)

Figure S7 TZOA3 Particle Count to reference concentration ratio and Relative Humidity (a) and Hourly Average FRM $PM_{2.5}$ concentration and TZOA3 Particle Count stratified by Relative Humidity (b)
